# Earthquake Exposure and Post-traumatic Stress Among Nepalese Mothers After the 2015 Earthquakes

**DOI:** 10.3389/fpsyg.2019.00734

**Published:** 2019-04-02

**Authors:** Ingrid Kvestad, Suman Ranjitkar, Manjeswori Ulak, Ram K. Chandyo, Merina Shrestha, Laxman Shrestha, Tor A. Strand, Mari Hysing

**Affiliations:** ^1^Regional Centre for Child and Youth Mental Health and Child Welfare, NORCE Norwegian Research Center, Bergen, Norway; ^2^Department of Child Health, Institute of Medicine, Kathmandu, Nepal; ^3^Department of Community Medicine, Kathmandu Medical College, Kathmandu, Nepal; ^4^Department of Research, Innlandet Hospital Trust, Lillehammer, Norway; ^5^Centre for International Health, University of Bergen, Bergen, Norway; ^6^Department of Psychosocial Science, Faculty of Psychology, University of Bergen, Bergen, Norway

**Keywords:** earthquake, disaster, PTSD, Nepal, mothers

## Abstract

**Introduction:** Nepal suffered from major earthquakes in April 2015 resulting in great damage to the society. The objective of the current study is to describe the earthquake exposure, the impact on family’s daily life and the symptoms of post-traumatic stress disorder (PTSD) and their association in Nepalese mothers 20 months following the earthquakes.

**Methods:** In a clinical trial in Bhaktapur, Nepal, 558 mothers responded to an inventory on earthquake exposure and the Impact of Event Scale – Revised (IES-R) 20 months after the earthquakes. In multiple linear regression models, we estimated the associations between the earthquake exposure and the impact on the families’ life and the IES-R score.

**Results:** Over 60% reported that the earthquakes had a great deal of negative impact on their family’s life. In 4.7%, close family members died, and in 10.5%, family members were injured. 24% had IES-R scores indicating PTSD symptoms within clinical concern or a possible diagnosis. Lower levels of education were associated with higher scores on the total IES-R. Mothers who report that the earthquakes had a great deal of negative impact had higher total IES-R scores [9.8, 95% CI (5.9, 13.6)] compared to mothers that reported no such negative impact. Mothers with family members who were killed had higher IES-R scores [3.6, 95% CI (1.6, 5.5)] than those with no family members who died. Mothers assisting in rescue efforts had lower IES-R scores [2.8, 95% CI (0.8, 4.8)] than those not assisting.

**Conclusion:** Our study demonstrates high levels of exposure to traumatic events, large negative impact on the everyday life of the families, and a high level of PTSD symptoms. There was a consistent and graded association between the exposure variables and PTSD symptoms. The large impact of the earthquakes on these Nepalese mothers underscore the importance of awareness of mental disorders following major natural catastrophes for marginalized families.

## Introduction

On April 25, 2015 Nepal was hit by a 7.9 Richter scale earthquake followed by another 7.4 Richter scale earthquake a few weeks later. The earthquakes resulted in about 9000 deaths, at least 23,000 injured and up to 3.5 million people displaced due to the destructions (Basnyat et al., 2015). Recovery and rebuilding efforts after the earthquakes have been slow (Adhikari et al., 2016). Reconstructions of damaged health facilities were still lacking 1 year after the earthquakes, and mental health services were particularly affected (Sherchan et al., 2017). A blockade at the southern border shortly after the earthquakes, led to a 6-month long fuel and essential commodities crisis. This situation hampered the recovery process even further, and may have had additional negative effects on the situation of the population (Lamichhane, 2015; Budhathoki and Gelband, 2016; Sharma et al., 2017). Four months after the earthquakes, population-based studies indicate a high rate of mental disorders among direct survivors in earthquake affected districts (Luitel et al., 2016; Kane et al., 2017). The long-term psychological consequences in the Nepalese population, however, are not yet known.

Major earthquakes are known to give rise to long-term consequences on mental health. The prevalence of mental disorders reported in studies after natural disaster are, however, within a wide range (Onder et al., 2006; Cairo et al., 2010; Cenat and Derivois, 2014). A recent systematic review and meta-analysis on the mental health consequences after earthquakes, presents incidence of post-traumatic stress disorder (PTSD) from 1.2 to 82.6%, with a combined incidence of 23.7% (Dai et al., 2016), while a more recent meta-analysis identified prevalences ranging from 4.1 to 67.07% (Tang et al., 2017). The impact of natural disasters on mental health depend upon several pre-, peri- and post-disaster factors (Tang et al., 2017). For instance, the extent of PTSD symptoms following earthquakes are demonstrated to be higher in people from low socioeconomic situations (Kilic and Ulusoy, 2003; Priebe et al., 2009; Cenat and Derivois, 2014; Carmassi et al., 2018), in individuals with low social support (Cairo et al., 2010; Ke et al., 2010; Feder et al., 2013) and in females (Kilic and Ulusoy, 2003; Priebe et al., 2009; Dell’Osso et al., 2011c, 2013; Carmassi et al., 2014; Cenat and Derivois, 2014). Furthermore, the risk of PTSD has repeatedly been shown to be associated with the severity of exposure to the disaster, with direct survivors at most risk (Dell’Osso et al., 2013; Piccardi et al., 2017). And finally, there are several post-earthquake characteristics such as loss of houses and property conflicts that may augment the impact of the crisis for the ones involved (Chen et al., 2007; Zhang et al., 2011). For those who participate in rescue efforts after earthquakes, however, the prevalence of PTSD symptoms have been shown to be lower than for other survivors (Galea et al., 2005). The extent to which the earthquake experience leads to long-term consequences on mental health depend upon these co-occurring factors, and thus early awareness of these risk-factors is important in order to plan early treatment intervention in the aftermaths of natural disasters (Roncone et al., 2013; Piccardi et al., 2017).

Low to middle income countries are the hardest-hit regions for natural disasters, with women and children most affected (Nour, 2011). Thus, mothers who experienced the major earthquakes in Nepal, may be an especially vulnerable group for long-term mental health problems (Khanal et al., 2015). The consequences of PTSD symptoms in this group may also have more severe consequences since maternal distress may influence the development and growth of children both during pregnancy, and after birth through impaired nurturing care (Surkan et al., 2011; Britto et al., 2016). Furthermore, in low to middle income countries characterized by poor social security systems, large catastrophes such as major earthquakes pose a particular burden on families that are already marginalized due to low socio-economic status. Thus, understanding the impact of natural disasters on women with caregiving responsibilities in vulnerable populations in low-income countries is of importance. In an ongoing clinical trial in Bhaktapur, Nepal we have collected information on the level of exposures to the earthquakes, the impact of the exposure on the family’s daily life across multiple life domains and maternal PTSD symptoms in 558 mothers. The objective of the current study is to describe the exposures and the impact from the earthquakes on important areas of life indirectly affected by the earthquakes, the level of PTSD symptoms as measured by the Impact of Event Scale – Revised (IES-R) (Weiss, 2007) in 558 Nepalese mothers, and their association, 20 months following the earthquakes.

## Materials and Methods

### Study Setting and Participants

Data for this study was collected within a randomized controlled trial on infant nutrition, growth and neurodevelopment (CT ClinicalTrials.gov: NCT02272842) (Strand et al., 2017). The study targeted 600 mildly stunted infants with close follow up for 12 months and was carried out in Bhaktapur municipality and the adjoining districts close to the capital city Kathmandu. Nepal is among the world’s least developed countries, and one-third of the population lives below the poverty line (Shrestha et al., 2014). Bhaktapur is a relatively homogenous community where most of the families are traditionally engaged in agriculture. Ownership of land and houses are key socio-economic (SES) indicators.

In April 2015, when the study was in its early beginning, Nepal was struck by large earthquakes and the district of Bhaktapur, one of the most densely populated districts of Nepal, was severely affected. After a few weeks, the study team managed to continue the enrollment for the trial and reached the total sample size of 600 enrolled children in January 2017. At the time of the earthquakes, some of the mothers had already given birth to the study children (from newborns up to 11 months), some of the mothers were pregnant, while others were not yet pregnant with the child who would be enrolled in the main clinical trial.

In order to gain more knowledge on the influence of large natural catastrophes on the daily life of vulnerable families in low income countries, we decided to include additional questionnaires on the earthquake exposure experienced by the families in the clinical trial, as well as a widely used assessment tool for PTSD symptoms. The current study thus benefits from being in the framework of a high-quality clinical trial with attrition kept to a minimum through close follow up to the families from the study team.

### Procedure

Approximately 20 months after the earthquakes, from December 2016 through January 2017, a group of trained field workers visited the homes of the enrolled children and asked the mothers questions on earthquake exposures, the perceived impact of the earthquakes on the daily life, and PTSD symptoms. These questionnaires were an addition to the original study protocol, and the visits were performed independent of the original study procedures. From the baseline of the original study we had information on demographics and the socio-economic situation of the families, such as parental level of education, occupation and standard of living.

For the original study, after providing thorough information on the study procedures to the parents (usually the mother), we obtained a written informed parental consent or parental thumbprint from those who were illiterate, for the children to participate in the study. For this addition to the study, we gathered a new informed written consent from the mothers. The study was approved by the Nepal Health Research Council and the Norwegian Regional Committee for Medical and Health Research Ethics (REK Sør-Øst), amendments were performed and approved particularly for this addition to the study.

### Instruments

The earthquake exposure questionnaire was constructed for the current study to assess exposures during the earthquakes, negative experiences from the earthquakes and the impact of the earthquakes on the families’ daily life. The included traumatic exposures during the earthquakes were seeing someone who were injured or killed, whether they assisted in rescue efforts and if family members (relatives or close family) were trapped, injured, or killed. We also asked the mothers to rate the perceived threat to their own life, or the life of someone close to them during the earthquake (“not at all,” “Some/Just a little,” and “a great deal (Nordanger et al., 2013). The impact of the earthquakes on the family’s daily life was assessed by a five-point scale: “Not at all” (0); “a little” (1); “some” (2); “quite a lot” (3); and “to a large extent” (4) on economy, food security, employment and the state of their houses. The questionnaire was based on previous traumatic exposure questionnaires (Rubin et al., 2007; Nordanger et al., 2013) and on discussions in the team of investigators and field workers with experience from the study field. The instrument was piloted among 29 women in the study population.

The IES-R is a measure of symptomatic status of PTSD after specific traumatic events (Weiss, 2007). The questionnaire measures the degree of distress related to a traumatic event in the past 7 days on a Likert scale, and provide a total score (range 0–88), and three subscale scores; Avoidance (range 0–32), Intrusion (range 0–32), and Hyperarousal (range 0–24). The scale is originally from the United States, but is widely used worldwide (Galea et al., 2005; Dai et al., 2016). We translated the IES-R from English to Nepalese according to recommendations (Weiss, 2007). First, we made a translation from English to Nepalese in our team of experienced psychologists and medical doctors. The back-translation was conducted by a person fluent in English and Nepalese and not otherwise affiliated with the study. The original version and the back-translated version was compared by our team of psychologists and medical doctors, and the final questionnaire was piloted on a sample of 12 women in the study population. The standardized alpha for the total scale was 0.87, and ranged from 0.50 to 0.80 in the subscales, indicating acceptable internal consistency. The IES-R scale is not a diagnostic tool of PTSD and consequently there are no recommended cut offs for a PTSD diagnosis. In the current study we use a score of 24 or more to indicate that PTSD should be a clinical concern (Asukai et al., 2002), and above 33 to indicate a probable PTSD diagnosis (Creamer et al., 2003). These cut offs have shown good psychometric properties when validated against a diagnostic interview.

### Statistical Analyses

All forms were manually checked for inconsistencies and double entered. Data are presented as numbers (N)/percentage (%) and as mean/standard deviation (SD). We used multiple linear regression models to examine the association between the independent variables (exposure and negative impact variables) and the dependent variable (total IES score), adjusting for potential predefined confounders (mother’s age and level of education). The exposure variables were: family members died, injured or trapped; believed own life/life of someone close was in danger; assisting in rescue efforts, while the negative impact variables were: negative impact on your family’s life; on food security; on employment; on economy; and state of the house. In linear regression models, we also examined the associations between SES factors (maternal/paternal level of education; number of rooms in the home; ownership of house; ownership of land; and living in joint vs. nuclear families) and the total IES-R score. In these models we adjusted for mothers age. For the models including maternal and paternal level of education, we used the contrast command in STATA to estimate the trend.

**FIGURE 1:**
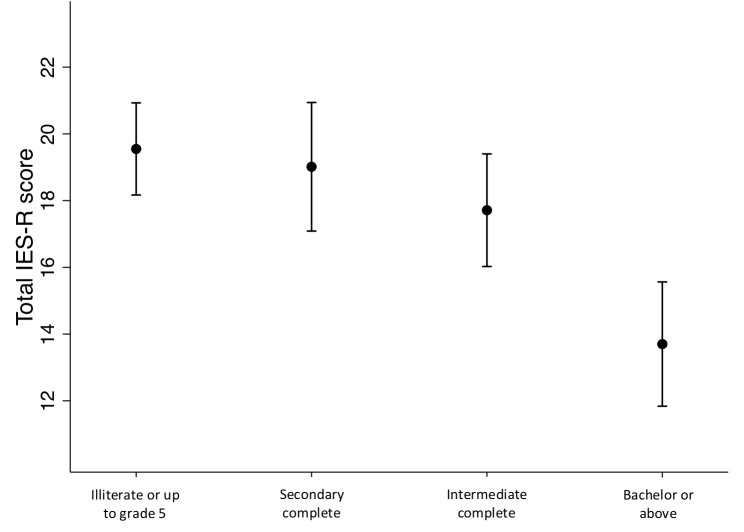
Estimated marginal means of the total Impact of Event – Revised scores by the mothers’ level of education. Adjusted for age.

**FIGURE 2:**
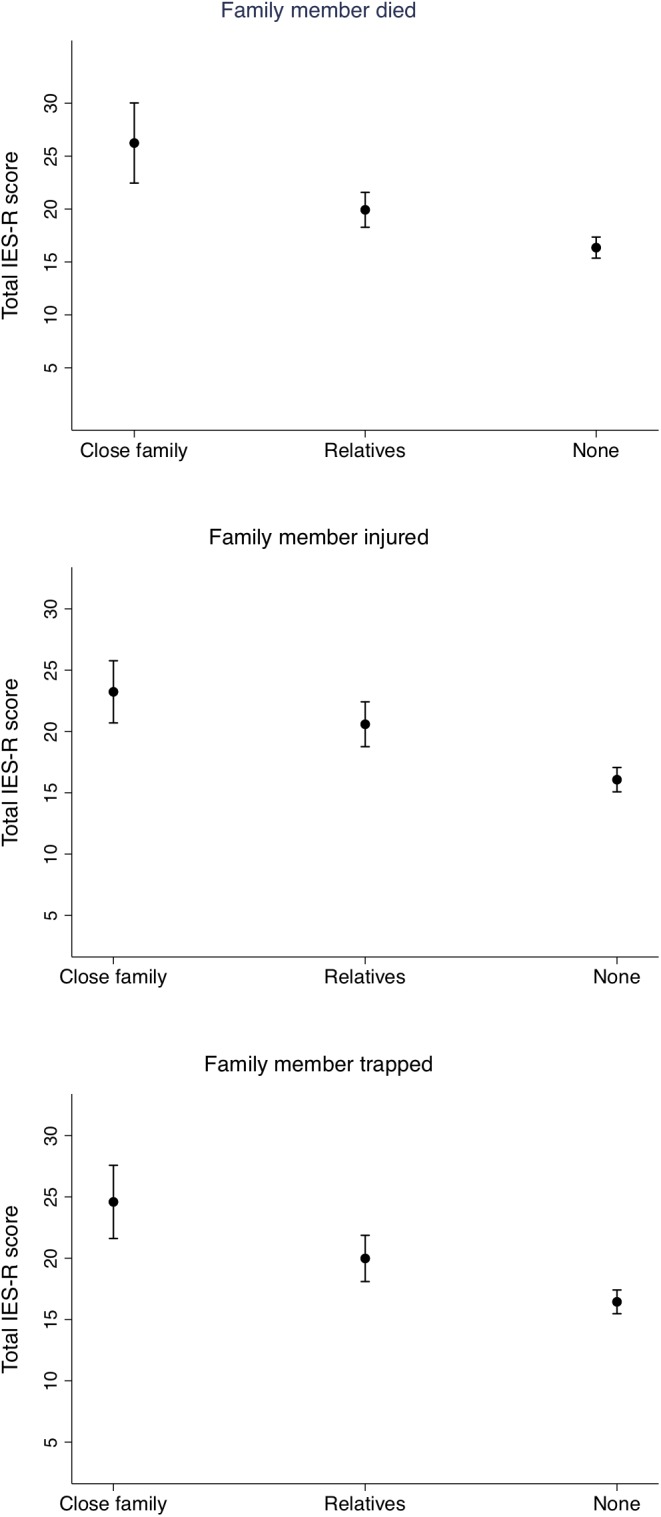
Estimated marginal means of the total Impact of Event – Revised score by whether close family or relatives died, were injured or trapped during the earthquakes. Adjusted for mother’s age and level of education.

**FIGURE 3:**
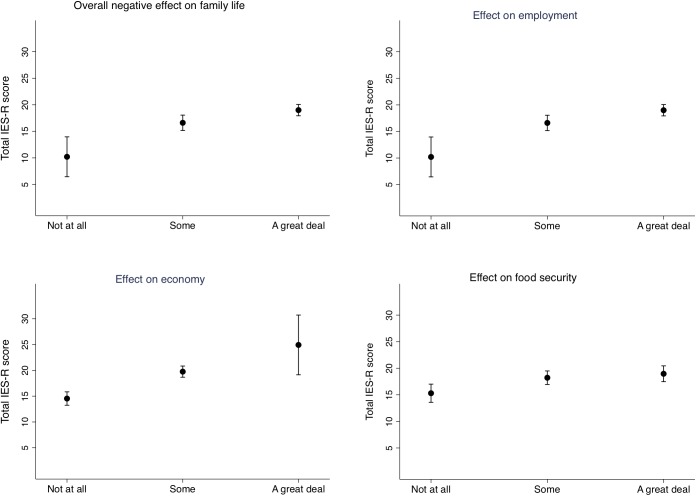
Estimated marginal means of the total Impact of Event – Revised score by the degree of influence of the earthquake exposure on the families’ daily life. Adjusted for mother’s age and level of education.

In the linear regression models, the total IES-R score was used on a continuous scale, while the main exposure variables were included on a three-point scale: “not at all”(0 from the original questionnaire), “some (merged 1 and 2 from the original questionnaire) and “a great deal” (merged 3 and 4 from the original questionnaire), or in the educational categories: Illiterate or up to 5th grade (0–5 years), Secondary complete (5–10 years), Intermediate complete (10–12 years), and Bachelor or above (12 years and more). The effect estimates are expressed as regression coefficients. In the figures, we display estimated marginal means of the total IES-R scores by the level of education in the mother (Figure 1) and by the level of exposure in selected exposure variables (Figure 2, 3). We also conducted a correlation analysis between the exposure, negative impact and SES factor variables and the total IES-R score by Spearman’s rho. Data was analyzed using STATA version 15 (StataCorp. College Station, TX, United States).

## Results

We were able to include 558 mothers from the original sample of 600. Six women did not answer the earthquake exposure questionnaire, and 552 mothers are included in the regression models. The demographic characteristics of the participants are shown in Table 1. Table 2 shows the correlation between the exposure, negative impact and the SES variables, and the scores on the IES-R. The IES-R score is correlated with the exposure and negative effect variables, but with the educational level variables only among the SES factors.

**Table 1:** Demographic characteristics of the 558 Nepalese mothers.

	*N*	Prevalence (%)
**Demographic features**		
Age, mean (SD)	27.6	4.6
Level of education:		
a. Illiterate up to grade 5	205	36.7
b. Secondary complete	105	18.8
c. Intermediate complete	137	24.5
d. Bachelor or above	112	20.1
Occupation:		
a. Not working/Agriculture	345	61.8
b. Carpet worker	15	2.7
c. Daily wage earner	70	12.5
d. Services	68	12.2
d. Working abroad	59	10.6
Husband level of education:		
a. Illiterate up to grade 5	196	35.2
b. Secondary complete	122	21.9
c. Intermediate complete	137	24.6
d. Bachelor or above	33	5.9
Socio-economic status		
Family staying in joint family	280	50.1
Family residing in rented house	260	46.5
1 to 2 rooms in the household	307	54.9
Family own land	272	48.7
2 or more children below age 5 in the home	158	28.3

**Table 2:** Correlation coefficient^1^ between total Impact of Event Scale–Revised score and the exposure, negative impact and socioeconomic factor variables in 552 families in Bhaktapur, Nepal.

**Exposure**	Family member died	−0.2071^∗∗∗^
	Family member injured	−0.2148^∗∗∗^
	Family member trapped	−0.1993^∗∗∗^
	Believed own life/life of someone close was in danger	0.1742^∗∗∗^
	Assisting in rescue efforts	0.0860^∗^
**Negative impact**	Negative impact on your family’s life overall	0.1914^∗∗∗^
	On food security	0.1327^∗∗^
	On employment	0.1914^∗∗∗^
	On economy	0.2828^∗∗∗^
	State of the house	0.1303^∗^
**Socio-economic factors**	Level of education	−0.1882^∗∗∗^
	Husbands level of education	−0.1843^∗∗∗^
	Number of rooms in the house	−0.0665
	Ownership of house	−0.0664
	Ownership of land	−0.0227
	Living in a joint vs. nuclear family	−0.0489

*^1^Spearman‘s Rho. ^∗^p < 0.05, ^∗∗^p < 0.01, and ^∗∗∗^p < 0.001.*

### Earthquake Exposures and the Negative Impact on the Family’s Daily Life

More than 60% (339) reported that the earthquakes had “a great deal” of negative effects on their family’s life. The specific aspects of life in which the earthquakes had a negative effect are shown in Table 3. As a result of the earthquakes, 4.7% reported that close family members died, 10.5% (58) that family members were injured, and 7.6% trapped.

**Table 3:** Earthquake exposure and the negative impact in 552 families in Bhaktapur Nepal.

		Not at all		Some		A great deal	
		*N*	%	*N*	%	*N*	%
Any negative effects of the earthquakes on your family’s life?		28	5.1	185	33.5	339	61.4
Which aspects of life did the earthquakes affect							
	Food security	134	24.3	240	43.5	178	32.2
	Employment	63	11.5	241	44.1	243	44.4
	Health related issues	289	52.4	180	32.6	83	15
Economic impact of the earthquake on the family life		219	39.9	319	58.1	11	2

		**No**		**Relatives**		**Close family**	

Family members died		383	69.4	143	25.9	26	4.7
Family members injured		382	69.2	112	20.3	58	10.5
Family members trapped		403	73	107	19.4	42	7.6

More than 75% (420) reported that they believed to “a great deal” that their own, or the life of someone close to them was in danger during the earthquakes. More than 40% (241) of the mothers saw someone get seriously injured, and approximately 50% (277) saw someone die. 76% reported that they participated in rescue efforts.

Half of the families had intact houses after the earthquakes, while 20% reported that their house was partially destroyed and 30% that their house had totally collapsed or was not usable. Of the total sample, only 2% reported that they did not move after the earthquakes, while 16.8% (92) reported that they have stayed more than 4 places during the past 20 months. More than 80% reported that they had been involved in conflicts regarding property arrangements after the earthquakes (Table 4).

**Table 4:** Earthquake exposures and the negative impacts in 552 families in Bhaktapur Nepal.

	*N*	%
**Adverse experiences during the earthquakes**		
Saw someone get seriously injured	241	43.6
Saw someone dead	277	50.3
Believed own life/life of someone close was in danger		
Not at all	37	6.6
Some	95	17.2
A great deal	420	76.3
Assisted in rescue efforts?	419	75.9
**Post-earthquake housing issues**		
State of house after the earthquake		
Intact	273	49.5
Partially destroyed	114	20.6
Collapsed/not usable	165	29.9
Current residence		
own damage home	110	19.9
temporary house	71	12.8
own intact home	149	26.9
rent	211	38.1
Others (relatives)	12	2.3
Places stayed after the earthquake		
Not moved	11	2
2 places	238	43.5
3 places	206	37.7
more than 4 places	92	16.8
Post-earthquake conflicts due to property arrangement?	460	83.8

### Post-traumatic Symptoms

Scores on the IES-R are shown in Table 5. Of the mothers, 17.1% had scores indicating that PTSD is a clinical concern, while 7.1% had scores indicating a probable PTSD diagnosis.

**Table 5:** Scores on the Impact of Event Scale–revised in 558 Nepalese mothers 20 months after the Earthquakes.

IES-R Score	Mean (*SD*)	Range
Total score	17 (9.9)	0–52
Intrusion	6.7 (4.7)	0–25
Avoidance	6.5 (4.3)	0–22
Hyperarousal	4.6 (2.9)	0–15

	***N***	**%**

PTSD is a clinical concern (score 24–32)	96	17.1
Probable PTSD diagnosis (score >33)	40	7.1

### Demographics and Socio-Economic Factors and the IES-R Score

The estimated marginal means of the total IES-R scores by the level of education in the mothers are shown in Figure 1. Compared to being illiterate or up to grade 5, a level of Bachelor or above was associated with a 5.8 [95% CI (8.2, 3.5), *P* < 0.001] reduced IES-R score. There was no significant difference between being illiterate or up to grade 5 and the other categories, but a significant trend (*p* < 0.001) toward a graded association between the level of education and the total IES-R scores. The same pattern of associations was observed between the husbands’ educational level and the mothers IES-R scores. There were no significant associations between the other demographic and SES variables and the total IES-R score.

### Associations Between Earthquake Exposures and Post-traumatic Stress Symptoms

Figure 2 displays the estimated marginal means of the total IES-R score by whether close family or relatives died, were injured or trapped during the earthquakes. In the adjusted regression models, mothers who had close family members that died had on average 9.9 [95% CI (6.0, 13.8), *P* < 0.001] points higher IES-R scores than mothers who reported that no family members were killed. Mothers with relatives that died had on average 6.2 [95% CI (2.1, 10.3), *P* < 0.003] points higher IES-R score. Similar patterns are seen for mothers with relatives or family members who were trapped or injured. In the adjusted models, mothers who reported that they believed that their own life, or the life of someone close to them was in danger both “some” and “a great deal” had scores that were on average 5.5 [95% CI (1.6, 9.3), *P* = 0.005] and 5.3 [95% CI (1.9, 8.6), *P* < 0.001] points higher than the mothers that felt “not at all” threatened. Finally, assisting in rescue efforts after the earthquakes was associated with 2.8 [95% CI (0.8, 4.8), *P* = 0.006] points lower IES-R scores compared to mothers who did not assist in such efforts.

### Associations Between Negative Impact of the Earthquakes on the Family’s Life and the IES-R Score

The estimated marginal means of the total IES-R score by the degree of impact on the families’ daily life are shown in Figure 3. Compared to no negative impact on the family’s life, experiencing “some” impact was associated with a score that was 6.4 [95% CI (2.4, 10.4), *P* = 0.002] points higher, while experiencing “a great deal” of impact was associated with 8.8 [95% CI (4.9, 12.7), *P* < 0.001] points higher total IES-R score. The negative impact is seen aspects of life such as on food security, employment and economy (Figure 3). Compared to having a house that was intact after the earthquakes, having a partially destroyed house was associated with a 3.1 [95% CI (0.8, 5.3), *P* = 0.007] higher total IES-R score, while having a collapsed house was associated with a 1.9 [95% CI (−0.1, 3.9), *P* = 0.057] increased score.

## Discussion

Results from the present study demonstrate the heavy burden set on the Nepalese mothers after the 2015 earthquakes in the densely populated and heavily affected city of Bhaktapur, Nepal. The mothers report a high negative impact from the earthquakes, both in terms of exposure to traumatic events and the negative impact on their everyday life. Twenty months following the earthquakes, our results demonstrate high levels of PTSD symptoms. In general, our results suggest that the more traumatic experiences and negative impact on the family’s life the mothers described, the more symptoms of PTSD in the mothers. In addition, we find that lower levels of education in both mothers and their husbands were associated with increased symptoms of PTSD, while participating in rescue efforts after the earthquakes were associated with less PTSD symptoms.

### Earthquake Exposure

The mothers in the present study report of severe, widespread and long-lasting impact of the earthquakes, including loss of family members, damaged and collapsed houses forcing the family to move several times, and negative impact on their practical family life in areas such as economy, food security and employment. Our findings are consistent with other studies describing the long-lasting impacts of earthquakes (Onder et al., 2006; Kolbe et al., 2010; Dell’Osso et al., 2011a; Feder et al., 2013). After the earthquakes in Nepal, there are reports that the rebuilding has been slowed by a range of challenges related to technical, social, and political processes (Adhikari et al., 2016; Meding et al., 2017). In the current study, more than 80% of the mothers report that their family were involved in conflicts due to property arrangements following the earthquakes. Difficulties in living arrangements has previously been presented as an important post-earthquake predictor for poor mental health (Tang et al., 2017). These devastating and long-lasting consequences also mirror the low-income countries vulnerability in both the scope of the destruction and in the long rebuilding period that is seen after the disaster.

### Prevalence of Post-traumatic Stress Symptoms

Approximately 24% of the mothers report PTSD symptoms that give rise to clinical concern or indicating a probable diagnosis. This is about the same level as the estimated incidence of PTSD after earthquakes in a recent meta-analysis, however, somewhat lower than the mean level among woman (Dai et al., 2016). A direct comparison is difficult, due to methodological differences across studies including time since the earthquake, the severity and exposure to the earthquakes and the measures of PTSD (Galea et al., 2005). Furthermore, the IES-R does not have established cut offs, and the prevalence may vary accordingly. While other studies have used lower cut offs (Dai et al., 2016) which would have increased the prevalence of PTSD in the current study, we have used 24 and 33 to indicate clinical symptoms and a probable diagnosis (Asukai et al., 2002; Creamer et al., 2003). This uncertainty of prevalence is accentuated by the fact that the IES-R has not been formally validated in the current population. However, with these reservations in mind, our findings do suggest high levels of PTSD symptoms. These high levels give rise to concern given the scarcity of mental health facilities in the Nepalese health care system. In spite of a mental health policy developed in 1997, the mental health services in Nepal are still limited to certain areas and groups, and were therefore unable to meet the demands after the earthquakes (Luitel et al., 2015; Sherchan et al., 2017). Following the earthquakes, there has been a strategic response to improve the mental health services and increase the preparedness for future disasters (Sherchan et al., 2017; Chase et al., 2018). As demonstrated in a study of 513 Nepalese adults 4 months following the earthquakes, more measures of mental health in addition to PTSD could have yielded a broader picture of the mental health burden in the current study (Luitel et al., 2016). In the previous study, few met the threshold for PTSD, however, more than 30% suffered from depression and anxiety and 20% from alcohol use disorder.

### Earthquake Exposure and the Negative Impact and Symptoms of Post-traumatic Stress

Exposure variables including the death of close family and the belief that own, or the life of someone close was in danger, were consistently related to higher PTSD symptoms in the present study. This is in line with the significant predictors of PTSD after earthquakes in recent meta-analyses (Dai et al., 2016; Tang et al., 2017). The pattern is also similar to the results from a study of young mothers after the Sichuan earthquake in China, where both the mother’s earthquake exposure and SES was associated with the level of PTSD symptoms (Qu et al., 2012). Furthermore, studies from the L’Aquila earthquake demonstrate that the degree of negative exposure in terms of the proximity to the earthquake epicenter (Dell’Osso et al., 2013) and loss of family or friends (Dell’Osso et al., 2011b) were related to PTSD. The present study expanded beyond the direct exposure of the earthquake to the impact of the earthquakes on other important areas of life indirectly affected by the earthquakes, such as employment and food security. Our results thus demonstrate that there is a consistent and graded association between the impact of these more distal life areas and PTSD symptoms 20 months following the earthquakes, suggesting that the impact on major life arenas increase post-traumatic stress symptoms in these women.

The previously mentioned review discuss how SES is inconsistently associated with PTSD after natural disasters (Galea et al., 2005). This mirrors the results in the present study where illiteracy and educational level up to grade 5 in the mothers and their husband is related to more PTSD symptoms, while none of the other SES indicators such as family ownership and size of house yielded such associations. Thus, in the current setting, literacy may be more specifically related to long-term adaption following major disasters. Our findings are similar to the results from studies from other earthquake hit areas, such as in Turkey, Haiti and Italy, where low level of education is one important predictor for mental health disorders following the disaster (Kilic and Ulusoy, 2003; Priebe et al., 2009; Cenat and Derivois, 2014).

Participation in relief efforts is associated with less PTSD symptoms than in the mothers that did not participate in such efforts. This may be unexpected, since the 419 women who assisted in rescue work most likely had an increased exposure to traumatic events, and there are studies that indicate that personnel working in emergency situations are at higher risk of PTSD (Carmassi et al., 2018). Previous studies have found lower prevalence of PTSD symptoms among those participating in rescue efforts after earthquakes, however, (Galea et al., 2005). In contrast to the mothers in the present study who were also direct survivors, participants in rescue efforts are often not directly exposed to the disasters. It may be that contributing to the community gives sense of meaning and perceived control which is associated with good mental health (Ikizer et al., 2016). Our findings could also be a result of a selection process, favoring those who are less likely to develop PTSD symptoms.

### Limitations

IES-R is a questionnaire assessing symptomatic status of PTSD and has not been validated for a Nepalese setting. A validated diagnostic interview would have given additional diagnostic information that would have strengthened the study. Mono-informant bias may have affected the results given the reliance on self- report. Many of the women are illiterate and the questions were asked through trained field workers. Due to the sensitive nature of the questions, some may have underreported the symptoms. Another limitation is the lack of information on previous mental health and/or clinical PTSD diagnosis, and comorbid mental health issues as some conditions like bipolar disorders, anxiety disorders or alcohol and substance use disorders can favor the onset of PTSD or influence its course.

Although the IES-R specifically states that the questions are related to the earthquakes of 2015, we cannot be sure whether the PTSD-symptoms of these women are related to lifetime stressful events that occurred before and/or after the earthquakes, rather than the earthquakes themselves. Finally, a limitation to the current study is that our sample may not be representative of the total population as we targeted families with mildly stunted children which may have resulted in a sample of relative low SES families. Furthermore, the study was restricted to mothers as informants. Fathers and other members of the extended family are also important caregivers and it is a limitation that we have not included them in the present study. Future studies should include multiple time-points to measure the change in symptoms reported over time, and include other measures of mental health in addition to PTSD. The effect of the PTSD symptoms on the children of the exposed mothers as described by others (Cai et al., 2017), was beyond the scope of the present study and should be further explored in this population in order to understand the impact of major natural disasters on the development of children.

### Implications for Future Research and Clinical Practice

The results from the current study underscore the importance of awareness of mental health issues in the aftermath of natural catastrophes in marginalized groups in low-income countries. Given the limited access to mental health services in Nepal it is a valid concern whether those in need received relevant care. Large scale programs targeting mental health consequences on a community level could be indicative after natural disasters in low-income countries.

## Data Availability

The datasets generated for this study are available on request to the corresponding author.

## Author Contributions

IK, SR, MU, RC, MS, LS, TS, and MH conceived the research. SR, MU, and RC participated in the data collection. IK, TS, and MH analyzed the data. IK and MH drafted and finalized the manuscript. All authors read, made suggestions and approved the final manuscript. IK had full access to all the data in the study and had final responsibility for the decision to submit for publication.

## Conflict of Interest Statement

The authors declare that the research was conducted in the absence of any commercial or financial relationships that could be construed as a potential conflict of interest.
